# Aspects and outcomes of surveillance for individuals at high-risk of pancreatic cancer

**DOI:** 10.1007/s10689-024-00368-1

**Published:** 2024-04-15

**Authors:** Aleksander M. Bogdanski, Jeanin E. van Hooft, Bas Boekestijn, Bert A. Bonsing, Martin N. J. M. Wasser, Derk C. F. Klatte, Monique E. van Leerdam

**Affiliations:** 1 Department of Gastroenterology and Hepatology, Leiden University Medical Center, Albinusdreef 2, 2333 ZA Leiden, The Netherlands; 2 Department of Radiology, Leiden University Medical Center, Leiden, The Netherlands; 3 Department of Surgery, Leiden University Medical Center, Leiden, The Netherlands; 4 Department of Gastrointestinal Oncology, Netherlands Cancer Institute, Amsterdam, The Netherlands

**Keywords:** Pancreatic ductal adenocarcinoma, Pancreatic cancer surveillance, High-risk individuals, Early detection

## Abstract

Pancreatic ductal adenocarcinoma (PDAC) is a leading cause of cancer-related deaths and is associated with a poor prognosis. The majority of these cancers are detected at a late stage, contributing to the bad prognosis. This underscores the need for novel, enhanced early detection strategies to improve the outcomes. While population-based screening is not recommended due to the relatively low incidence of PDAC, surveillance is recommended for individuals at high risk for PDAC due to their increased incidence of the disease. However, the outcomes of pancreatic cancer surveillance in high-risk individuals are not sorted out yet. In this review, we will address the identification of individuals at high risk for PDAC, discuss the objectives and targets of surveillance, outline how surveillance programs are organized, summarize the outcomes of high-risk individuals undergoing pancreatic cancer surveillance, and conclude with a future perspective on pancreatic cancer surveillance and novel developments.

## Background

Pancreatic ductal adenocarcinoma (PDAC) is a major health problem with a growing incidence [1]. It is anticipated that it will become the second-leading cause of cancer-related deaths by 2030 [2]. PDAC has a poor prognosis with a 5-year survival rate of less than 10% [3]. While surgical resection is the only curative treatment, 80% of PDACs are unresectable at presentation [4, 5]. This underscores the necessity for early detection. Nonetheless, pancreatic cancer screening of the general population is not feasible due to the relatively low incidence of this disease, with 13.3 cases per 100,000 individuals per year [6]. Even a highly accurate test would result in many false-positively diagnosed individuals, who would be subjected to unnecessary harm including operation, excessive psychological burden following from screening, and would lead to high costs [7, 8]. Accordingly, pancreatic cancer screening of the general population is currently not recommended by guidelines [9–12]. While that is the case, pancreatic screening may prove more effective when applied to subpopulations at an increased risk for PDAC [13].

Interestingly, it is known that up to 10% of individuals with PDAC have an underlying genetic predisposition [14]. The incidence of PDAC among these high-risk individuals (HRIs) is higher compared to that of the general population, making surveillance more appropriate in that subpopulation. Although data on long-term screening results are recently starting to be published, several questions persist. It remains unclear whether surveillance results in an increased resection rate of PDAC, avoids an excessive number of unnecessary surgeries and improves survival outcomes compared to no surveillance. Therefore, the aim of this review is to present a comprehensive overview of pancreatic cancer surveillance among HRIs. We will touch on who is considered to be at high-risk of PDAC, outline the objectives and targets of surveillance, describe how surveillance programs are organized, report outcomes and conclude with a future perspective on pancreatic cancer surveillance.

## Selection of individuals at high-risk

HRIs are individuals with an increased risk of PDAC based on family history or a germline pathogenic variant (PV) status and require a minimum lifetime risk of PDAC of ≥ 5% [15]. Notably, there is variation in the cumulative incidence of PDAC among different germline PV carriers which underscores the complexity of selecting HRIs [16]. With some of the high-risk PVs being present in the *PRSS1/SPINK1* gene carrying a lifetime risk of 7.2–53.3% [16]. This is followed by the *STK11/LKB1* gene (11–36%) and the *CDKN2A/p16* gene (19%) [16]. Adding to the complexity is the observation that PDAC may manifest at a younger age in certain carriers [17, 18].

In certain germline PV carriers the mutation status alone may not pose sufficient PDAC risk to be included in the surveillance program, therefore, family history of PDAC is also taken into account [19]. However, there is limited literature on the impact of positive family history on PDAC risk in predisposition genes, such as *BRCA1/2, ATM* and *MLH1/MSH2/MSH6* [9, 10]. In fact, recent studies conducted on the impact of family history in individuals with PVs in *BRCA1/2* genes have found no association between a positive family history and an increased risk of PDAC [20, 21]. Additionally, these studies recommend that all individuals with PVs in the *BRCA* genes to be included in pancreatic cancer surveillance, regardless of family history [20, 21]. Further research is required to confirm this.

Due to the variations among germline PV carriers, distinct recommendations are made for the starting age of surveillance for each specific group of germline PV carriers [9, 12]. An overview of known PDAC predisposition genes with starting age recommendations per guideline is shown in Table 1.

**Table 1:** International guidelines starting age recommendations for pancreatic cancer surveillance per germline pathogenic variant

Pathogenic variant in	CAPS 2019 [9]		AGA 2020 [12]		AISP 2020 [11]		ASGE 2022 [10]	
	Family history	Starting age	Family history	Starting age	Family history	Starting age	Family history	Starting age
ATM (ataxia-teleangiectasia)	≥ 1 FDR	45 or 50^a^	≥ 1 FDR	50^a^	–	–	≥ 1 FDR or ≥ 1 SDR	50^a^
BRCA1 (HBOC)	≥ 1 FDR	45 or 50^a^	≥ 1 FDR	50^a^	≥ 1 FDR or ≥ 1 SDR	40^c^	–	50^a^
BRCA2 (HBOC)	≥ 1 FDR	45 or 50^a^	≥ 1 FDR	50^a^	≥ 1 FDR or ≥ 1 SDR	40^c^	–	50^a^
CDKN2A/p16 (FM)	–	40^a^	–	40	–	30	–	40^a^
FPC	≥ 1 FDR who in turn also has ≥ 1 FDR	50 or 55^a^	≥ 2 affected relatives	50^a^	≥ 1 FDR and ≥ 1 SDR	45^a^	≥ 2 FDR	50^a^
STK11/LKB1 (PJS)	–	40^a^	–	35	–	30	–	35^a^
MLH1/MSH2/MSH6 (Lynch syndrome)	≥ 1 FDR	45 or 50^a^	≥ 1 FDR	50^a^	≥ 1 FDR or ≥ 1 SDR	40^c^	≥ 1 FDR or ≥ 1 SDR	50^a^
PALB2 (HBOC)	≥ 1 FDR	45 or 50^a^	≥ 1 FDR	50^a^	≥ 1 FDR or ≥ 1 SDR	40^c^	–	50^a^
PRSS1/SPINK1 (hereditary pancreatitis)	–	40^b^	–	40	–	40^c^	–	40

– not applicable, *AGA* American gastroenterological association, *AISP* Italian association for the study of the pancreas, *ASGE* American society for gastrointestinal endoscopy, *CAPS* international cancer of the pancreas screening consortium, *FAP* familial adenomatous polyposis, *FDR* first-degree relative, *FM* familial melanoma, *FPC* familial pancreatic cancer, *HBOC* hereditary breast- and ovarian cancer syndrome, *PJS* Peutz–Jeghers syndrome

^a^Or 10 years younger than the youngest affected blood relative

^b^Or 20 years after the first pancreatitis attack

^c^Or 5 years younger than the youngest affected blood relative

Individuals who do not meet the criteria for a known PDAC-associated inherited cancer syndrome but have two or more affected first-degree relatives (FDR) are characterized as a distinct group due to their strong family history. This group is referred to as ‘familial pancreatic cancer’ (FPC). Individuals with FPC, having three affected FDRs exhibit a standardized incidence ratio for PDAC of 32.0, while those with two affected relatives have a ratio of 6.4 [9, 22]. To our knowledge, there is currently no literature available on the distinct risks of PDAC in individuals with FPC, who have affected second-degree relatives (SDR). More research should be done to better understand the influence of a positive family history on the risk for PDAC among HRIs.

## Objective and targets of pancreatic cancer surveillance

The primary objectives of pancreatic cancer surveillance are the early detection of PDAC, thereby improving the PDAC-related survival, and the detection of high-risk precursor lesions with the goal to decrease the incidence of PDAC [9]. According to the current guidelines, detection of stage I pancreatic cancer and high-grade dysplasia (HGD) precursor lesions, including pancreatic intraepithelial neoplasia (PanIN)-3 and intraductal papillary mucinous neoplasm (IPMN) with HGD, is considered a successful surrogate outcome marker of surveillance [9, 12]. In accordance with American Joint Committee on Cancer guidelines, stage I pancreatic cancer is confined to the pancreas, with no evidence of tumor spread to the lymph nodes, and distant sites [23]. It is further divided into stage IA (T1N0M0) and IB (T2N0M0) based on size, with stage IA pertaining to tumors measuring 0–2 cm and stage IB including tumors ranging from 2 to 4 cm [23].

### Pancreatic intraepithelial neoplasia (PanIN)

PanINs are microscopic lesions in the pancreas, measuring less than 5 mm in diameter [24]. They are characterized by metaplasia of small ducts in the pancreas, replacing the normal cuboidal epithelium [25]. PanINs are classified into three grades, ranging from low-grade (PanIN1 and PanIN2) to high-grade dysplasia (PanIN3), with neoplastic progression driven by the accumulation of genetic alterations and, therefore, also associated with age [24]. PanIN3 was previously referred to as ductal carcinoma in situ due to its resemblance to invasive carcinoma [25]. PanIN lesions are recognized as precursors for invasive PDAC and are particularly common in the elderly [25]. In a study by Longnecker et al. [26], 86.4% of individuals at autopsy were found to have 1–43 PanINs. On average, the pancreases containing these PanINs harbored a mean of eight lesions. However, only a subset of these lesions will progress to invasive PDAC, with the likelihood of progression dependent on the grade [27]. The lifetime progression rate of PanIN-1 to PDAC is estimated at 1.3–1.5%, PanIN-2 to PDAC at 5.04–5.94% and PanIN-3 to PDAC at 28–33% [27]. As a result, surveillance is considered successful only when high-grade PanINs are resected. Additionally, the significance of numerous PanINs remains unclear. A study of Kiemen et al. [28] has found that PanINs are interconnected and form extensive networks of lesions rather than being solitary lesions. Further research is needed to understand the behavior of these PanINs and to identify additional factors that influence their progression into PDAC in the context of germline mutations, aiming to prevent the onset of PDAC and to avoid unnecessary surgical procedures.

### Intraductal papillary mucinous neoplasm (IPMN)

IPMNs are macroscopic lesions that develop within the pancreatic ductal system [29]. These lesions are characterized by their cystic nature, mucin secretion and are less common than PanINs [29]. Studies on HRIs indicate that cystic lesions, particularly branch-duct (BD) type IPMN, are the most commonly diagnosed abnormalities during pancreatic cancer surveillance [30–37]. This occurrence is not unusual, considering the high prevalence of IPMNs, which can reach up to 11.3–25% among the general population aged ≥ 55 years and increases with age [38, 39]. Additionally, there is evidence suggesting that among HRIs, the cumulative incidence of IPMNs is even higher, exceeding 46% [40]. Like PanINs, IPMNs are recognized as precancerous lesions and only a minority progress into PDAC [29]. The ones that do progress, advance from low-grade IPMN to PDAC in approximately 6 years. [41] The rate of progression is associated to the location of the lesion [41]. Based on their location, IPMNs can be categorized into three groups: main duct (MD), branch duct (BD), and mixed type (MT) involving both locations [42]. Individuals with MD and MT-IPMNs have a relative or absolute indication for surgery in the presence of specific risk factors, as outlined in the European evidence-based guidelines [43].

Also, BD-IPMN do not immediately warrant surgery according to guidelines due to their lower malignant potential [42, 43]. Surgical indications for BD-IPMNs include the presence of jaundice or high-risk features, such as dilatation of the main pancreatic duct or enhancing mural nodules [43, 44]. Notably, a study revealed that there is an elevated likelihood of IPMNs progressing to PDAC among HRIs [40]. Therefore, despite acknowledging the low progression rate of BD-IPMN, ongoing surveillance of these lesions remains essential in HRI [42]. In relation to this, a multicenter observational study among HRIs found that BD-IPMNs without any worrisome features or high-risk stigmata showed no difference in the risk of developing PDAC after 5 years of surveillance compared to the general population, depending on age and cyst size [45].

## Surveillance methods

Individuals who meet the criteria for HRIs are enrolled in a pancreatic cancer surveillance program. According to current recommendations, intermittent longitudinal imaging, including magnetic resonance imaging (MRI)/magnetic resonance cholangial pancreatography (MRCP), and/or endoscopic ultrasound (EUS) is suggested [9]. Occasionally, computed tomography (CT) may be considered when both modalities are contraindicated, such as in the presence of metal implants or claustrophobia [46, 47]. Nonetheless, CT is not recommended as a first-line test due to the radiation accumulation [9]. Furthermore, due to the limited evidence for pancreatic cancer surveillance, guidelines recommend that surveillance and yield evaluation should be performed in specialized centers only [9].

### MRI

Imaging of the pancreas is complex due to the shape of the organ and the central location of the organ in the abdomen. The combination of time-consuming data acquisition and motion due to respiration, gastrointestinal peristalsis, cardiac activity and vascular pulsation can lead to image degradation and motion artifacts [48]. High-quality scans with minimal number of artifacts are needed to accurately assess the pancreas and to enable early detection of pancreatic cancer. Ideally, a 3.0 Tesla scanner is used due to its superior signal-to-noise ratio, leading to higher quality images, however, pancreatic imaging can also be performed using 1.5 Tesla scanners [49]. The MRI sequences that are commonly used in clinical practice to assess the pancreas, include T1-weighted gradient-echo, T2-weighted axial and coronal sequences, and MRCP [49]. All these sequences can be performed within thirty minutes [49]. The T1-weighted MRI images are useful for assessing pancreatic fat and hemorrhage within inflammatory collections [49]. While an increase in pancreatic fat has been linked to the development of PanINs, the routine clinical assessment of pancreatic fat remains constrained [50, 51]. It should be noted that PanINs are microscopic lesions that cannot be directly detected with current imaging techniques [24]. However, literature shows that these lesions are correlated with pancreatic fatty infiltration, which can be seen on imaging and can therefore be used to identify PanINs [52, 53]. The T2-weighted MRI images are usually performed to illustrate the pancreatic ducts and potential cysts [49]. The high pancreatic fluid contrast in this sequence allows for lesion characterization [49]. Beyond the aforementioned MRI sequences, there are additional sequences available for the evaluation of PDAC, however, these are outside the scope of this review. The Pancreatic Cancer Early Detection (PRECEDE) consortium, which consists of a considerable number of institutions where HRIs for PDAC are undergoing surveillance at forty sites across North America, Europe and Asia, has developed a consensus statement to standardize MRI surveillance [54]. This statement includes the specification of which MRI sequences should be used and how to report findings in these individuals [54].

### EUS

Besides MRI, EUS is used to evaluate the pancreas. In contrast to MRI, establishing clear-cut recommendations for EUS imaging is challenging [55]. The difficulty stems from the complexity of standardizing EUS procedures as these are strongly dependent on operator expertise, which is a crucial factor in ensuring successful EUS procedures [56]. Clear imaging of the pancreas must first be achieved before any assessment can occur. EUS is used to identify small lesions or as an adjunct for further testing or confirmation when abnormalities are detected on cross-sectional imaging [55]. While EUS can detect lesions up to < 10 mm, small or deeply located lesions may be difficult to visualize [57]. Moreover, obese individuals with increased adipose tissue may limit the visibility of EUS and the presence of gas or other obstructions may limit probe accessibility, making assessment of the pancreas more challenging [58].

In addition to pancreatic evaluation, EUS is also used to diagnose lesions through EUS-fine-needle aspiration (FNA) and fine-needle biopsy (FNB). This procedure is identical to EUS, except that a needle is inserted through the working channel of the endoscope to obtain samples from lesions. A meta-analysis examining the diagnostic accuracy of FNA and FNB in solid pancreatic masses showed that FNB demonstrates higher accuracy compared to FNA, with rates of 87% versus 80%, respectively [59].

### MRI or EUS

While there is a lack of consensus regarding the variations in diagnostic yield between MRI and EUS, some studies suggest that MRI may be a better option for evaluating cystic lesions, whereas EUS may be more preferred for detecting solid lesions and parenchymal changes [60–62]. This preference could be explained by the high soft-tissue contrast of MRI, facilitating the assessment of cystic lesions, and the high spatial resolution of EUS, enabling a more comprehensive evaluation of solid lesions and parenchymal alterations [63]. An important advantage of MRI is the ability to evaluate lesion progression over time by comparing consecutive images. Moreover, the preference for the MRI may stem from its less invasive nature and standardized procedures, in contrast to the more operator-dependent nature of EUS [54, 56, 57]. This is even more apparent in the detection of small abnormalities, a task that should be performed by experienced endoscopists. Nevertheless, it should be emphasized that expertise is equally important for MRI, as a thorough examination of the pancreas should be conducted by experienced pancreatic radiologists to ensure that significant lesions are not missed [54].

In summary, the two modalities complement rather than replace each other.

### Surveillance program

**Fig. 1:**
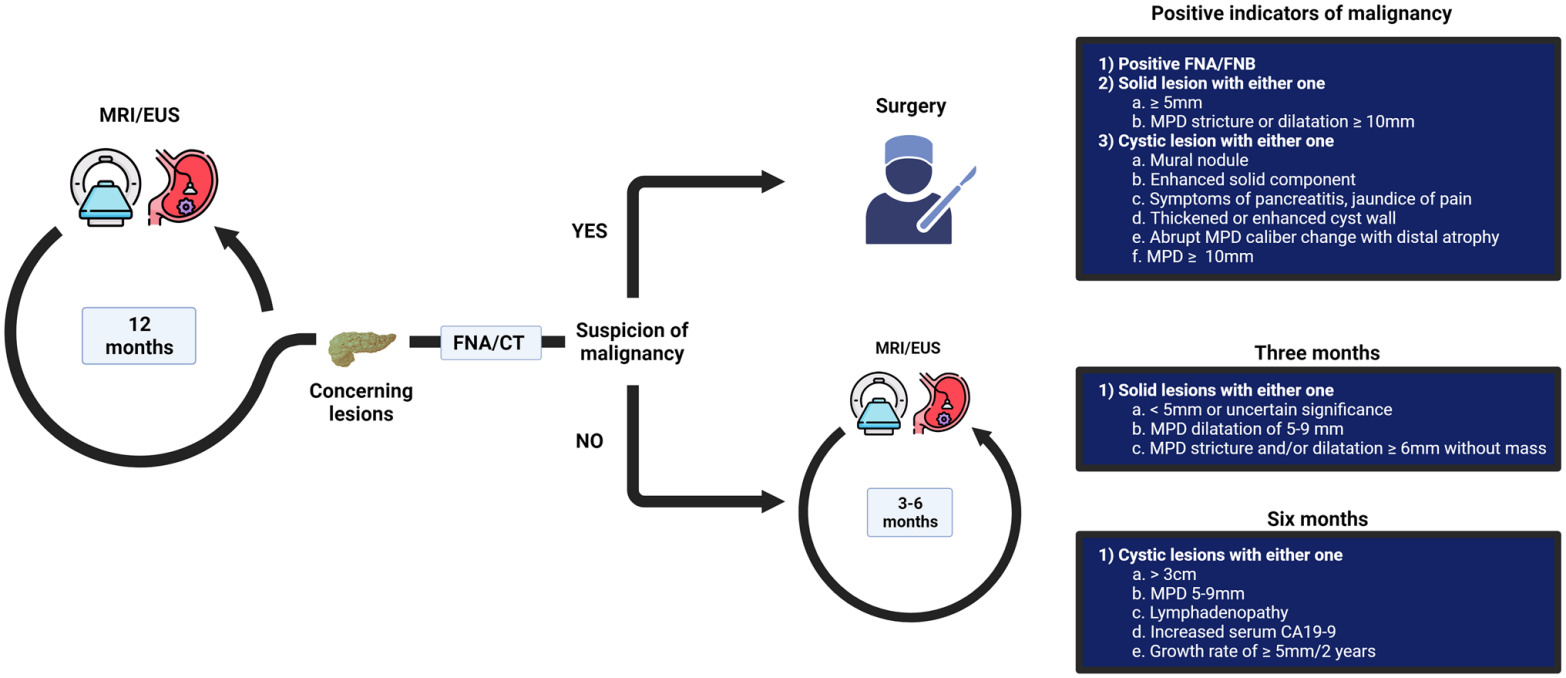
Overview of pancreatic cancer surveillance in high-risk individuals based on CAPS guidelines [9]. High-risk individuals undergo annual surveillance. Upon detection of new concerning lesions, EUS with FNA/FNB or CT is performed to assess the potential malignancy of the lesion. If malignancy is suspected, surgery is recommended. The positive indicators of malignancy are shown in the adjacent table. Conversely, when malignancy is not indicated, individuals transition into a shortened surveillance protocol. The shortening of the surveillance interval depends upon the characteristics of the lesion and is illustrated in the corresponding tables. *CT* computerized tomography, *FNA* fine needle aspiration, *FNB* fine needle biopsy, *MPD* main pancreatic duct dilatation. Created with BioRender.com

Surveillance typically involves annual longitudinal imaging by MRI and/or EUS. If any concerning lesions are found, such as cysts with worrisome features, solid lesions or main pancreatic duct (MPD) stricture and/or dilatation ≥ 6 mm without a mass, further assessment is conducted using either EUS with FNA/FNB or CT to evaluate the suspicion for malignancy. If the detected lesion is suspected to be malignant, surgery is recommended [9]. For lesions that are not suspected of malignancy, individuals may undergo a shortened surveillance interval to closely monitor the lesion. The determination of the surveillance interval is dependent upon the characteristics of the lesion [9]. When lesions remain stable or diminish over time, individuals may return to the 12-month surveillance interval. However, there are no guidelines specifying exactly when one can return to the regular interval or can even discontinue surveillance. Figure 1 provides a brief overview of a pancreatic cancer surveillance program, including positive indicators of malignancy and characteristics of lesions suggesting a three- or six-month surveillance interval.

### Biomarkers

As of today, carbohydrate 19-9 (CA19-9) is the only FDA-approved biomarker for diagnostics of PDAC. However, it is crucial to underscore that CA19-9 is employed exclusively when PDAC is suspected in a clinical setting and is not utilized in surveillance settings, where the a priori likelihood of PDAC varies and influences both positive and negative predictive values. Studies have evaluated the added value of CA19.9 in cancer screening trials and pancreatic cyst surveillance, demonstrating high specificities of up to 99% for PDAC [64, 65]. Nevertheless, these studies also detected significantly low sensitivities, reaching as low as 17% [64, 65]. The ability of CA19-9 to reliably identify PDAC is influenced by several conditions, including liver diseases, pancreatitis, cholangitis, as well as pulmonary and gynecologic diseases, which may lead to potential false-positive results for PDAC [66, 67]. Moreover, studies suggests that certain individuals lack the Lewis antigen and produce minimal to no CA19-9 [68, 69]. Consequently, these individuals may not exhibit a sufficiently elevated CA19-9 level to meet the standardized threshold of > 37–40 U/mL for effective PDAC detection [70]. The variability in CA19-9 levels is, therefore, also dependent on the genetic variations present in individuals. In a related development, Dbouk et al. [71] personalized the CA19-9 cut-off threshold for PDAC based on these genetic variations. This adjustment led to an improvement in AUROC from 0.84 without the personalized cut-off to 0.92 with the personalized cut-off [71]. While these results are promising, further validation is needed before CA19-9 with personalized cut-offs will be implemented in the clinical setting. Lastly, several studies are being conducted on biomarkers that could improve the early detection of PDAC and potentially complement the surveillance programs in the future, but these are beyond the scope of this review [72].

## Outcomes of high-risk individuals undergoing pancreatic cancer surveillance

### Meta-analyses

Several studies have investigated the outcomes of HRIs undergoing pancreatic cancer surveillance. A meta-analysis by Signoretti et al. [60] examined the diagnostic yield of pancreatic cancer surveillance programs for successful target lesions, defined as PanIN-3, high-grade IPMN and any resectable PDAC with R0 pathology. This meta-analysis included all studies encompassing individuals with FPC or other high-risk germline PV carriers who underwent pancreatic cancer surveillance based on MRI and/or EUS. In total 16 studies were included, involving 1588 HRIs, of which 1043 (66.3%) had FPC, 243 (15.4%) had *CDKN2A* and 140 (8.9%) had HBOC (*BRCA1, BRCA2* and *PALB2*). The diagnostic yield, evaluated separately for different HRI groups, was defined as the pooled prevalence of successful target lesions. This is the number of successful target lesions detected over the entire follow-up period, divided by the total number of individuals undergoing surveillance. The study demonstrated a diagnostic-yield for FPC (3%), *PRSS1/SPINK1* (hereditary pancreatitis; 4%), *CDKN2A* (FM) (5%), *BRCA1/2* and *PALB2* (HBOC) (6.3%), and *STK11/LKB1* (Peutz–Jeghers syndrome; PJS; 12.2%). Moreover, this study showed that five successful target lesions were found per 1000 person-years. This suggests that two hundred HRIs are needed to screen (NNS) to find one successful target lesion within this composition of HRIs.

The meta-analysis by Corral et al. [73] has confirmed these findings and reported an incidence rate for successful target lesions of 7.4/1000 person-years and a NNS of 135. This meta-analysis included 19 studies, with in total 7085 individuals and defined successful yield as individuals with PanIN3, HGD-IPMN or non-metastatic PDAC. This study also calculated the NNS for specific HRI groups, with a NNS of 250 for a PV in *BRCA* genes, 130 for *PRSS1/SPINK1* (hereditary pancreatitis), 71 for *STK11*/*LKB* (PJS) and 51 for a PV in the *CDKN2A* gene. However, it is necessary to point out that both meta-analyses consist of overlapping studies, and this may partially explain the comparable results. Additionally, it is worth emphasizing that these findings include successful target lesions of PanIN3, HGD-IPMN and R0 or non-metastatic PDAC, extending beyond the criteria outlined in the CAPS guidelines, which specifically focus on stage I PDAC [9]. However, given that individuals with PDAC beyond stage I also benefit from surgery, these two meta-analyses effectively illustrate the pooled diagnostic yield for pancreatic cancer surveillance across different HRI groups [74].

### Findings from recent studies

The meta-analyses mentioned above included studies up to the year of 2017. Since then, additional studies have been published on the outcomes of HRIs in pancreatic cancer surveillance. Below, we will outline these studies and their corresponding findings.

In 2018, Canto et al. [75] conducted a study on a surveillance cohort of 354 individuals in the United States. The cohort mainly consisted of individuals with FPC (n = 297) and the remaining 57 individuals were carriers of a genetic PV. Notably, 41 of these individuals carried a PV in *BRCA1/BRCA2,* or *PALB2* genes and the entire cohort included only individuals with at least six months of follow-up. The mean age of the cohort was 56.4 years and the median follow-up was 5.6 years. During the entire follow-up period a total of 10 (2.8%) PDAC cases were identified with a resection rate of 90% and a three year survival of 85%. Considering the diagnostic yield of the surveillance program, 1 (10%) stage I PDAC, 6 HGD-IPMNs and 4 PanINs-3 were detected during surveillance. In this study, 23/354 (6.5%) individuals underwent surgery for suspected malignancy, only to find out that the lesions were benign. All PDAC cases occurred in individuals with FPC, whose age ranged from 46 to 79 years old. This contrasts with the findings of Overbeek et al. [76], who have found no PDAC cases in the FPC cohort, suggesting a possible increase in the starting age of these individuals. The reasons for this disparity might be clarified by the uncertainty regarding whether genetic testing was conducted on individuals with FPC in the study from Canto et al. [75] Potential PV carriers within the Canto et al. [75] cohort might have increased the risk of PDAC. In fact, a more recent and unrelated evaluation of 5/10 cases of the Canto cohort revealed three cases of true FPC with PDAC, one individual with PV in *ATM* and one with a PV in the *BRCA2* gene [77].

In 2022, Overbeek et al. [76] have published a study on the yield of HRIs undergoing surveillance. This study included a Dutch cohort of 366 individuals, consisting of 201 FPC and 165 PV carriers. All FPC individuals underwent genetic testing and were proven not to be carriers of a known PV. Notably, none of the FPC individuals developed PDAC, and all 10 (2.7%) PDAC cases were identified in the carriers of a germline PV, including 7 in *CDKN2A* carriers, 2 in *STK11/LKB1* and 1 in a *BRCA2* carrier. In total 3 (30%) stage I cancers were detected and the resection rate for PDAC was 60%. Median survival for patients with PDAC was 18 months and was adversely influenced by 3 out of 10 cases. Moreover, 11/366 (3%) individuals in this study underwent surgery for a benign lesion. Based on these results with no PDAC cases in the FPC group, the authors speculate whether a higher starting age should be considered for FPC individuals. Furthermore, out of ten cases of PDAC, four (40%) were identified as interval cancers. Interval cancers are cases of pancreatic cancer that present in the period following a negative surveillance examination and before the next examination, which usually takes place after 12 months [78]. The number of interval cancers could be explained by the magnitude of PDAC risk, the chosen surveillance intervals and the limitation of the imaging modalities. A large study involving 2552 HRIs under surveillance revealed that in nearly half of the detected cases (46%), the median time to present a new lesion was 11 months. This implies that in half of the cases, PDAC or HGD will develop before the next scheduled annual surveillance examination. This is a concern, particularly when surveillance is delayed beyond the scheduled time [79]. Although the exact mechanism behind this rapid development and progression of PDAC is unknown, some studies suggest that chromothripsis may contribute to the rapid acquisition of mutations, particularly in the *BRCA* carriers [80–82]. Chromothripsis is a phenomenon characterized by multiple chromosomal rearrangements occurring in one or more chromosomal regions during a single event [80]. Nonetheless, little is still known about the tumorigenesis of PDAC in these individuals [83]. Whole genome sequencing studies may help to understand the carcinogenesis of these interval cases [84].

Another study, conducted in 2022, has examined the outcomes of pancreatic cancer surveillance in 347 *CDKN2A* germline PV carriers in the Netherlands [85]. The median enrollment age was 48.6 years and the median follow-up was 5.6 years. The study revealed an overall median survival of 26.8 months with an overall 5-year survival rate of 32.4%. In total 36 (10.4%) PDAC cases were detected. Out of all 36 detected PDAC cases, 12 (33.3%) were detected at stage I and 5 (13.9%) presented as interval cancers. Moreover, 27 (75%) individuals with PDAC underwent surgery. Throughout the entire follow-up, 7 out of 347 (2%) individuals underwent surgery for benign disease. In a separate study, the same cohort was used to investigate if surveillance was of added value compared to non-surveillance. So comparison was made between HRIs undergoing surveillance and PDAC diagnosis within the general population without surveillance. The study has shown that, even after accounting for potential lead times of 3, 6, 12, and 15 months, the surveillance cohort demonstrated a higher median overall survival compared to the non-surveillance cohort (23.9 months, 22.0 months, 19.7 months and 15.2 months, respectively, vs. 5.2 months) [86].

In 2023, a study was conducted on *BRCA1/2* PV carriers and involved a total of 180 individuals [87]. The cohort included 57 (31.7%) *BRCA1*, 121 (67.2%) *BRCA2* and 2 (1.1%) individuals with PV in other genes (*APC*, *MSH6* and *MSH2*). It is worth highlighting that a significant proportion (82%) of the study cohort consisted of Ashkenazi Jewish individuals, who typically carry specific founder allele mutations [88]. However, the impact of this on PDAC risk and survival outcomes compared to individuals with different PV within the *BRCA* genes is not known. In this study, individuals underwent annual surveillance using MRI and EUS, with the surveillance interval being adjusted as necessary in accordance with the CAPS guidelines [9]. All *BRCA1/2* carriers with at least one FDR/SDR with PDAC were enrolled in the surveillance program at the age of 45 or 10 years earlier than the age of the youngest relative diagnosed with PDAC. Additionally, 64 (35.6%) *BRCA1/2* carriers without a family history were included in the surveillance program due to their concern of developing PDAC. Over a median follow-up period of 48 months, a total of 4 (2.2%) cases of PDAC were detected, all of which occurred in the *BRCA2* carriers, with one individual also carrying the *MSH2* PV. Out of the 4 PDAC cases, only 1 (25%) was identified at stage I. The resection rate for all 4 cases was 75%, as one of them had distant metastasis and could not be resected. Additionally, during the entire surveillance period, 2/180 (1.1%) individuals underwent surgery for benign lesions. Unfortunately, median survival could not be determined with four cases. This could have been interesting information since *BRCA* carriers may benefit from selective poly (ADP-ribose) polymerase (PARP) inhibitors, potentially resulting in an increased overall survival [89].

Having provided in-depth summaries of the most recent studies, it is important to acknowledge that additional studies have been conducted on pancreatic cancer surveillance among HRIs. All of these studies, alongside the earlier discussed studies and those included in the meta-analyses are summarized in Table 2.

**Table 2:** Detailed overview of pancreatic cancer surveillance studies

Author, year	Country	Sample size	N male sex (%)	Follow-up, months (median)	Type of pathogenic variant	Age, start surveillance (median)	Detected PDACs	Resection rate	Successful yield	False-positive rate
Laish, 2023 [87]	Israel	180	112 (62.7%)	48	80 PV carriers - 57 *BRCA1* - 21 *BRCA2* - 2 *BRCA1/2*	55	4	75%	1 stage I	1.1%
Overbeek, 2022 [76]	The Netherlands	366	157 (42.9%)	63*	201 FPC 165 PV carriers - 96 *CDKN2A* - 45 *BRCA2* - 7 *BRCA1* - 2 *PALB2* - 9 *STK11/LKB1* - 5 *TP53* - 1 *ATM*	54*	10	60%	3 stage I	3%
Klatte, 2022 [85]	The Netherlands	347	146 (42.1%)	67.2	347 *CDKN2A*	48.6	36	75%	12 stage I	2%
Dbouk, 2022 [90]	United States	1461	517 (35.4%)	48	346 FPC 643 PV carriers - 269 *BRCA2* - 93 *ATM* *-* 69* CDKN2A* *-* 68* BRCA1* *-* 62 *PALB2* *-* 58 Lynch syndrome (not specified) *-* 18 *STK11/LKB1* *-* 6 more than one PV and positive family history 402 positive family history (1 FDR + ≥ 1 SDR) 65 other risk groups 5 positive family history of early onset PDAC in FDR	60.3*	10	80%	7 stage I 2 HGD 1 PanIN3	0.3%
Bartsch, 2021 [30]	Germany	295	Unknown	41	267 FPC 28 PV carriers (not specified)	55	2	100%	3 PanIN3	3.7%
Bar-Mashiah, 2020 [31]	United States	74	29 (39.2%)	31.2	50 FPC 24 PV carriers - 8 *BRCA1* - 13 *BRCA2* - 1 *ATM* - 2 Lynch Syndrome (not specified)	Unknown	1	Unknown	1 HGD- IPMN	Unknown
McNamara, 2019 [32]	United States	105	33 (31.4%)	Unknown	60 FPC 45 PV carriers - 24 *BRCA2* - 11 *BRCA1* - 1 *STK11* - 2 *ATM* - 1 *MSH6* - 1 *RAD50* - 5 *CDKN2A*	Unknown	2	0%	Unknown	Unknown
Paiella, 2019 [33]	Italy	187	87 (46.5%)	Unknown	165 FPC 22 PV carriers - 5 *BRCA1* - 5 *BRCA2* - 3 *CDKN2A* - 5 *STK11/LKB1* - 4 *PRSS1*	51*	5	40%	2 stage I 1 HGD-IPMN	0%
Sheel, 2019 [34]	United Kingdom	321	Unknown	24	268 FPC 53 PV carriers - 22 *BRCA2* - 5 *CDKN2A* - 4 FM (not specified) - 4 *STK11/LKB1* - 8 Lynch Syndrome (not specified) - 10 Other cancer syndromes; none with known causative mutations	Unknown	1	0%	None	0.9%
Barnes, 2018 [35]	United States	75	29 (39%)	Unknown	33 FPC 42 PV carriers - 18 *BRCA2* - 8 *ATM* - 6 *BRCA1* - 4 *CDKN2A* - 3 *PALB2* - 1 *MLH1* - 1 *PMS2* - 1 *STK11/LKB1*	56*	0	N/A	None	No resections
DaVee, 2018 [36]	United States	86	18 (20.9%)	29.8	86 PV carriers - 50 *BRCA2* - 14 *BRCA1* - 12 *p53* - 5 *STK11/LKB1* - 3 *MSH2* - 1 *ATM* - 1 *APC*	48.5	0	N/A	Unknown	Unknown
Canto, 2018 [75]	United States	354	168 (48%)	67.2	297 FPC 57 PV carriers - 10 *STK11/LKB1* - 4 *CDKN2A* - 41 *BRCA1/BRCA2/PALB2* - 1 Lynch Syndrome (not specified) - 1 *PRSS1*	56.4*	10	90%	1 stage I 6 HGD- IPMN 4 PanIN3	6.5%
Lachter, 2018 [37]	Isreal	123	70 (56.9%)	Unknown	29 FPC 17 PV carriers - 14 *BRCA2* - 3 *MSH6* 77 positive family history (not specified)	57*	1	100%	None	0.8%
Chang, 2017 [91]	Taiwan	303	116 (38.3%)	78	54 FPC 65 PV carriers - 47 *PRSS1* (24 confirmed by testing) - 17 *SPINK1* (7 confirmed by testing) - 1 *BRCA1/2* 184 positive family history	51.1*	7	100%	1 stage I	2%
Joergensen, 2016 [92]	Denmark	71	36 (50.7%)	60	40 FPC 31 PV carriers - 31 hereditary pancreatitis (not specified)	51.1*	2	100%	1 stage I	Unknown
Vasen, 2016 [93]	**Overlapping cases with** (Klatte et al. 2022)									
Harinck, 2015 [94]	**Overlapping cases with** (Overbeek et al. 2022)									
Del Chiaro, 2015 [95]	Sweden	40	16 (40%)	12.9*	32 FPC 8 PV carriers - 3 *BRCA2* - 1 *BRCA1* - 4 *CDKN2A*	49.9*	3	100%	1 Stage I	5%
Mocci, 2015 [96]	Spain	41	Unknown	Unknown	24 FPC 12 HBOC (not specified) 5 positive family history of early onset PDAC	Unknown	0	N/A	1 PanIN3	0%
Sud, 2014 [97]	United States	30	4 (13.3%)	Unknown	19 FPC 11 PV carriers - 7 *BRCA1/2* - 2 *STK11/LKB1* - 1 *CDKN2A* - 1 Lynch Syndrome (not specified)	51.3*	2	100%	1 stage I	3.3%
Potjer, 2013 [98]	**Overlapping cases with** (Klatte et al. 2022 + Langer et al. 2009)									
Al-Sukhni, 2012 [99]	Canada	262	98 (34%)	50.4*	159 FPC 93 PV carriers - 7 *STK/LKB1* - 2 Hereditary pancreatitis (not specified) - 68 *BRCA2* - 11 *CDKN2A* - 1 *BRCA1* 10 FDR with multiple cancers	54*	3	33.3%	None	0.8%
Canto, 2012 [100]	**Overlapping cases with** (Canto et al. 2018)									
Zubarik, 2011 [101]	United States	546	219 (40.1%)	Unknown	540 FPC 6 *BRCA* (not specified)	59*	1	100%	1 Stage I	0.4%
Ludwig, 2011 [102]	United States	109	31 (28.4%)	Unknown	93 FPC 7 *BRCA* (not specified) 9 positive family history of early onset PDAC	54*	1	100%	1 PanIN3	3.7%
Vasen, 2011 [103]	**Overlapping cases with** (Klatte et al. 2022)									
Poley, 2009 [104]	**Overlapping cases with** (Overbeek et al. 2022)									
Langer, 2009 [105]	Germany	76	Unknown	Unknown	74 FPC 2 *BRCA2*	Unknown	0	N/A	None	9.2%
Canto, 2006 [106]	**Overlapping cases with** (Canto et al. 2018)									
Canto, 2004 [107]	**Overlapping cases with** (Canto et al. 2018)									
Kimmey, 2002 [108]	United States	46	Unknown	Unknown	46 FPC	Unknown	0	N/A	Unknown	Unknown

Summary of studies conducted on HRIs undergoing pancreatic cancer surveillance. Successful yield includes stage I PDACs, PanINs-3 and HGD-IPMNs. The false-positive rate was defined as the surgical resection of benign lesions also including PanIn1, PanIN2, low- or intermediate-grade IPMNs

*FPC* familial pancreatic cancer, *HBOC* hereditary breast- and ovarian cancer syndrome, *HGD* high-grade dysplasia, *IPMN* intraductal papillary mucinous neoplasm, *N/A* not applicable, *PanIN* pancreatic intraepithelial neoplasia

## Future perspectives

Despite pancreatic cancer surveillance, PDAC cases are often diagnosed at a late stage (stage II or higher). A meta-analysis examining late-stage PDAC detection in HRIs undergoing pancreatic cancer surveillance showed that late-stage PDAC (1.7 per 1000 patient-years) represented a substantial proportion of the overall detection rate of PDAC (3.3 per 1000 patient-years) [109]. Therefore, novel methods should be explored for the early detection of PDAC.

Interestingly, a study conducted by Hoogenboom et al. [110] investigated the detectability of pancreatic cancer before PDAC diagnosis and has found the potential suspicion of a pancreatic mass in 50–70% of patients, with an abdominal CT scan for different indications, up to 3 years prior to PDAC diagnosis. Essentially, this suggests that a significant proportion of pancreatic cancers exhibit observable changes within this timeframe. However, not all changes in the pancreas are caused by PDAC. For instance, pancreatitis can mimic neoplastic progression and may be challenging to distinguish from PDAC [111]. Notably, recent research in murine models demonstrated the ability to differentiate between acute pancreatitis and PDAC using deuterium metabolic MRI [112]. In this study, deuterated glucose uptake and conversion into lactate, attributable to the Warburg effect, was evident in all PDAC cases but consistently non-existent in cases of pancreatitis [112]. While these findings are promising, they still have to be translated from murine models into humans. Many more potential radiomic biomarkers are being developed for PDAC, including the use of artificial intelligence [113].

Another interesting development is the use of a blood-based biomarker based on the glycosylation of proteins [114]. Acknowledging that a single gene can give rise to multiple proteins underscores the importance of exploring not only at the genetic level but also at the proteomic level in the search for potential biomarkers. In a study conducted by Vreeker et al. [115], an N-glycan profile was established for PDAC detection, demonstrating a sensitivity and specificity of 0.85–0.75 and 0.72–0.71, respectively. Nevertheless, these findings require further validation. Additionally, an evaluation is necessary to determine whether these N-glycans can effectively discriminate between PDAC and other benign pancreatic diseases, a challenge that persists in the routine clinical setting [116].

Moreover, while a pancreatic cancer surveillance program captures numerous cystic lesions, the limited ability to differentiate between malignant and benign cysts still leads to the unnecessary resection of benign cysts [117]. Genomic-based biomarkers show significant promise in addressing this challenge [118]. In a multicenter study investigating targeted next-generation sequencing using pancreatic cyst fluid, a sensitivity of 88% and a specificity of 98% were observed in detecting the presence of advanced neoplasia in these cysts [119]. Currently, surveillance relies solely on imaging, which is suboptimal [85]. In the future, complementing biomarkers will enhance the early detection of PDAC in HRIs.

Additionally, guidelines do not provide a recommendation regarding the age at which surveillance should be discontinued [9]. Establishing a stopping age is necessary to maintain the effectiveness of surveillance and minimize the burden. However, as every individual ages differently, with variations in fitness and health, determining a universal cutoff age is unrealistic. Reasons to discontinue surveillance, include limited life expectancy and potential risks associated with the procedures. Elderly are more likely to die from non-cancer-related causes and would subsequently no longer benefit from early PDAC detection [120]. Moreover, patients must meet a certain level of physical fitness to undergo procedures, otherwise early PDAC detection may not yield significant benefits, as disease treatment will become unfeasible. More research needs to be conducted in this field to offer guidance on what criteria to consider in assessing the added value of surveillance in older individuals or potentially develop a prediction model that can determine whether an individual will benefit from surveillance or not.

Lastly, due to the relative rarity of PDAC, conducting studies on early detection methods is challenging. Fortunately, the PRECEDE consortium and CAPS consortium gather extensive data from medical centers around the world and foster collaboration with institutions globally to facilitate the development of early detection methods [9, 57].

## Data Availability

No datasets were generated or analysed during the current study.
